# Microfiber-shaped building-block tissues with endothelial networks for constructing macroscopic tissue assembly

**DOI:** 10.1063/1.5109966

**Published:** 2019-11-13

**Authors:** Yuta Kurashina, Ryo Sato, Hiroaki Onoe

**Affiliations:** 1 Department of Mechanical Engineering, Faculty of Science and Technology, Keio University, 3-14-1 Hiyoshi, Kohoku-ku, Yokohama 223-8522, Japan; 2 Department of Materials Science and Engineering, School of Materials and Chemical Technology, Tokyo Institute of Technology, 4259 Nagatsutacho, Midori-ku, Yokohama 226-8503, Japan; 3 School of Integrated Design Engineering, Graduate School of Science and Technology, Keio University, 3-14-1 Hiyoshi, Kohoku-ku, Yokohama 223-8522, Japan

## Abstract

We describe a microfiber-shaped hepatic tissue for *in vitro* macroscopic tissue assembly, fabricated using a double coaxial microfluidic device and composed of cocultured Hep-G2 cells and human umbilical vein endothelial cells (HUVECs). The appropriate coculture conditions for Hep-G2 cells and HUVECs in the microfiber-shaped tissue were optimized by changing the thickness of the core and the cell ratio. The HUVEC networks were formed in the microfiber-shaped tissue following culture for 3 days. Using this microfiber-shaped tissue as a building block, two types of macroscopic assembled tissues were constructed—parallel and reeled tissues. In both tissue types, the connection of the HUVEC network across the adjacent microfiber-shaped tissues was established after 2 days, because the calcium alginate shell of the microfiber-shaped tissue was enzymatically removed. Our approach could facilitate the generation of complex and heterogeneous macroscopic tissues mimicking the major organs including the liver, kidney, and heart for the treatment of critically ill patients.

## INTRODUCTION

Concomitant with technological development in regenerative medicine, research focus on technologies to reconstruct *in vitro* three-dimensional (3D) tissues has been increasing considerably in recent years. The construction of scaffolds using decellularized tissues^1^ or biocompatible polymer materials^2^ has demonstrated the possibility of assembling *in vitro* reconstructed tissues applicable to regenerative medicine, including both *in vitro* and *in vivo* applications. One of the goals of regenerative medicine is to produce functional macroscopic tissues by combining these scaffolds with a wide variety of cells derived from stem cells, including induced pluripotent stem cells (iPSCs).^3^ To construct such functional macroscopic tissues *in vitro*, tissue engineering technologies that can assemble complicated and ordered structures composed of multiple types of cells are necessary. A top-down tissue engineering approach, involving a macroscopic scaffold seeded with cells, has been the gold standard in this field and has been used for simple *in vitro* tissue construction in clinical treatments.^4–9^ However, with regard to the generation of complex tissues containing multiple cell types, it is difficult to design and fabricate the detailed structures of such tissues using this top-down tissue engineering approach.

To overcome this difficulty, the focus has recently shifted toward a bottom-up approach to reconstruct complex functional tissues *in vitro.*^10^ In this approach, 3D macroscopic tissues are constructed via the assembly of a large amount of microscale tissues (typical tissue dimension: several tens to hundreds of a micrometer) as building blocks. By arranging building blocks of various shapes such as spheres,^11^ sheets,^12^ and fibers,^13^ it is possible to construct hierarchical and high-density tissues with endothelial networks necessary for long-term tissue stability owing to their function in the supply of nutrients and oxygen.^14^ Of the various types of these building blocks, microfiber-shaped building blocks^15–17^ have been the focus of extensive research as their shape characteristics and high handleability are expected to render them more suitable than other types of building blocks for the construction of hollow or oriented structures. To date, microfiber-shaped tissue construction via cells of the cardiomiocytes,^18^ and tendons^19^ has been reported. However, no reports of the successful construction of macroscale tissues containing networks of endothelial cells between tissues via the assembly of microfiber-shaped building blocks currently exist.

**FIG. 1.**
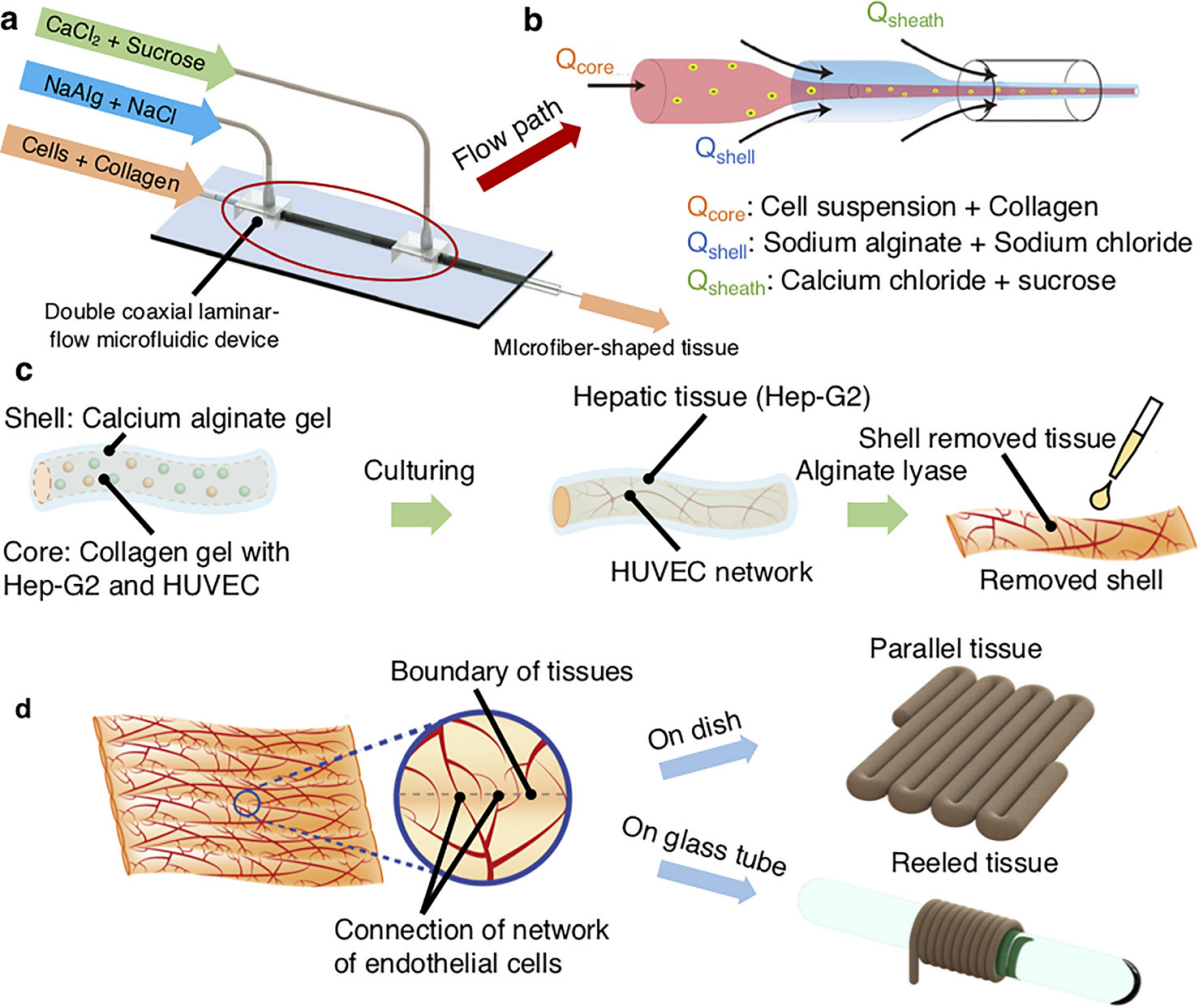
Concept of microfiber-shaped hepatic tissues with a network of endothelial cells. (a) The microfiber-shaped tissue was generated by using a double-coaxial microfluidic device. The microfiber-shaped tissue (core) was covered with a calcium alginate hydrogel (shell). (b) Flow schematic in the double coaxial microfluidic device. (c) Hep-G2/HUVECs encapsulated in the hydrogel microfiber were cultured for several days to form a microfiber-shaped hepatic tissue with HUVEC networks. The calcium alginate shell was removed by alginate lyase treatment in order to facilitate macroscopic tissue assembly. (d) The microfiber-shaped tissues were assembled and cultured for constructing macroscopic tissues: parallel tissues and reeled tissues.

Here, we report the experimental basis for the construction of a microfiber-shaped artificial tissue with endothelial networks aimed at constructing a macroscopic tissue construct with networks of endothelial cells (Fig. 1). This microfiber-shaped tissue was prepared by encapsulating the endothelial cells with the cells of the targeted tissue in a calcium alginate hydrogel tube to confine these cells inside the shell. Via the use of a double coaxial microfluidic device,^16^ the hydrogel microfiber containing cells could be quickly and easily fabricated at a scale of meters. The prepared microfiber could be cultured to construct a microfiber-shaped building block containing endothelial networks. In addition, the prepared microfiber-shaped building blocks could be assembled to facilitate the construction of macroscopic tissue constructs with networks of endothelial cells spread inside the tissue. In the present study, Hep-G2 cells were encapsulated into microfibers together with endothelial cells in the form of human umbilical vein endothelial cells (HUVECs) to construct a microfiber-shaped hepatic tissue with endothelial networks. We optimized the conditions for the construction of the HUVEC network by modulating the diameter of the microfiber-shaped tissue and the ratio of Hep-G2 cells to HUVECs. We also analyzed HUVEC network formation following the assembly of the microfiber-shaped tissue.

## RESULTS

### Formation of the microfiber-shaped tissue

**FIG. 2.**
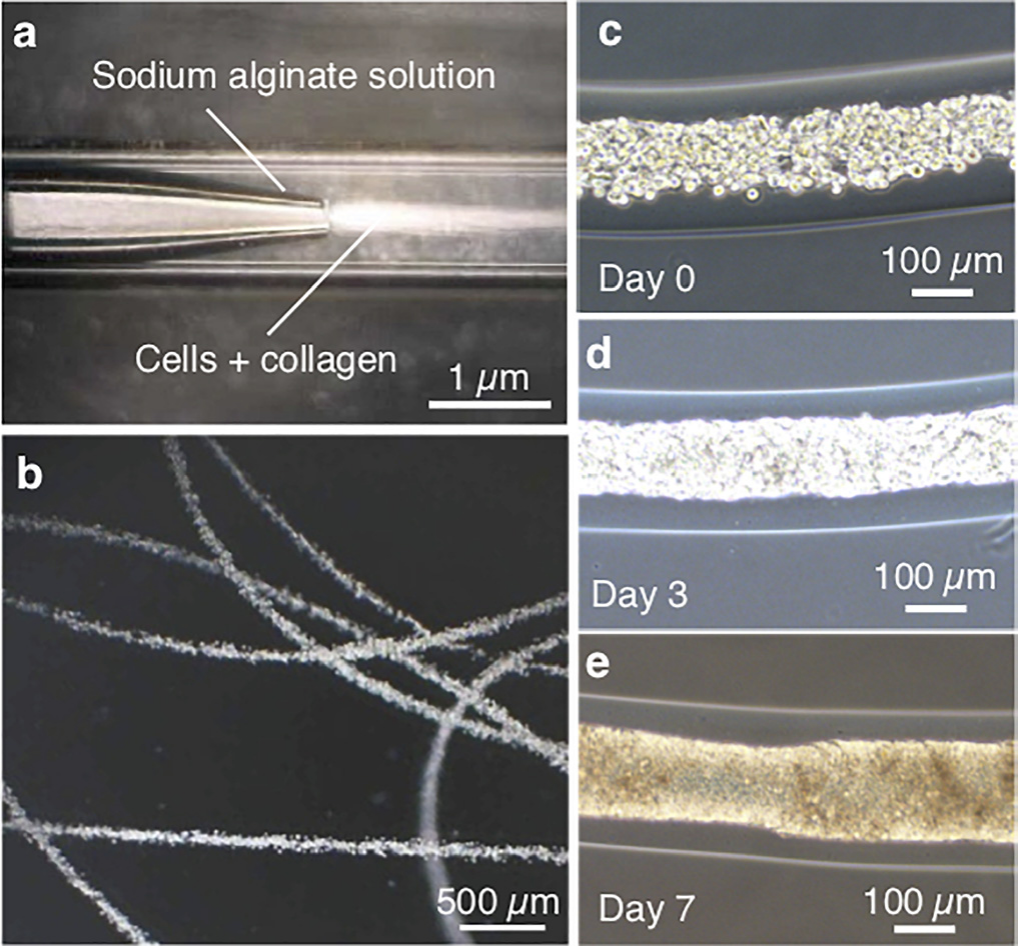
Generation of the microfiber-shaped tissue. (a) A core stream containing cells and collagen was surrounded by a shell stream of sodium alginate solution in the double coaxial microfluidic device. (b) Image of the cell-encapsulating hydrogel microfiber. (c)–(e) Hep-G2 cells and HUVECs forming a microfiber-shaped tissue during the culture.

We generated a hepatic microfiber-shaped tissue (HepG2 tissue) possessing a network of endothelial cells by using a core-shell hydrogel microfiber^16,20–22^ as a 3D culture platform. Our core-shell hydrogel microfiber was composed of a calcium alginate shell (diameter: ∼230 *μ*m) and a core of collagen gel containing Hep-G2 cells and HUVECs (diameter: ∼100 *μ*m) via continuous formation by using a double coaxial microfluidic device [Figs. 1(a) and 1(b)]. The typical length of the microfiber was approximately 1 m, and the diameter of the microfiber was uniform [Figs. 2(a) and 2(b)]. To observe the formation of the microfiber-shaped hepatic tissue with networked endothelial cells, we cultured cell-encapsulating hydrogel microfibers containing Hep-G2 cells and HUVECs (1:1 ratio) for 7 days [Figs. 2(c)–2(e), and supplementary Figs. 1(a)–1(h)]. The cells in the core [Fig. 2(c)] began to contact to one another and finally connected completely to form a microfiber-shaped tissue inside the tube-shaped calcium alginate shell on day 3 [Fig. 2(d)]. The shape of the tissue was stably maintained at day 7 [Fig. 2(e)], since the calcium alginate shell retained the structure without leaking the encapsulated cells.

### Formation of a HUVEC network in Hep-G2 microfiber-shaped tissues with different core diameters

**FIG. 3.**
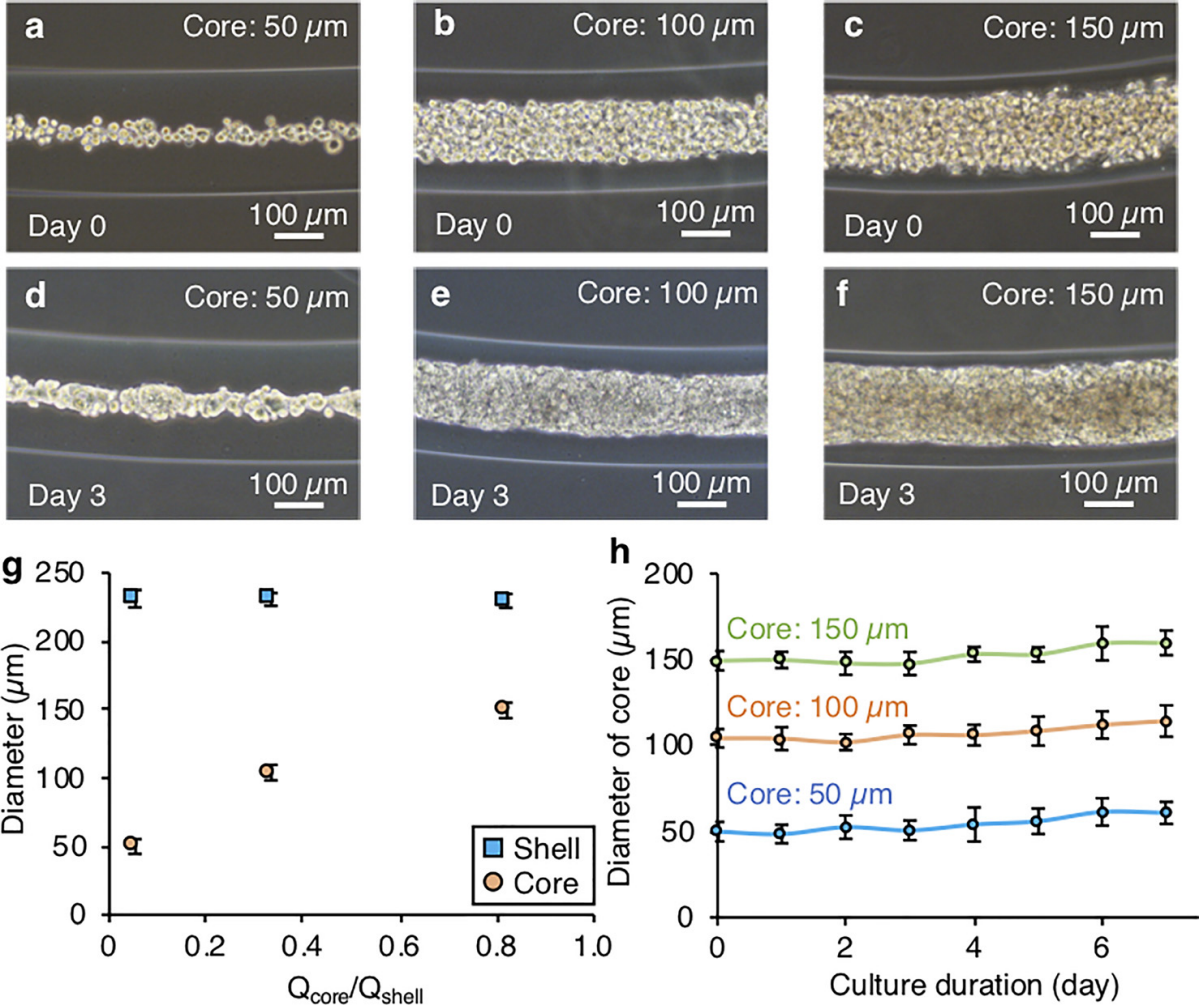
Formation of hepatic tissues of different core diameters. (a)–(f) Images of the microfiber-shaped tissues with different core diameters on day 0 (a)–(c) and day 3 (d)–(f). (g) The ratio of the core flow (Q_core_) and the shell flow (Q_shell_) for controlling the core diameter. (h) Changes in the tissue diameters in the core during the culture.

To examine how the growth of HUVEC networks is influenced by the surrounding culture environment, we first tested the network formation of HUVECs using microfiber-shaped tissues with different diameters. Figures 3(a)–3(f) shows the phase contrast images of the cultured microfiber-shaped tissues with three different core diameters (50 *μ*m, 100 *μ*m, and 150 *μ*m) on day 0 and day 3. The diameter of the core was varied by changing the ratio of the core and shell flow rates, *Q*_core_/*Q*_shell_, during the microfiber formation process [Fig. 3(g)]. For tissues of all diameters, the cells were initially dispersed in the collagen gel just after microfiber formation [Figs. 3(a)–3(c)]. After culturing, the cells gradually adhered to each other and microfiber-shaped tissues with different tissue diameters were formed on day 3 [Figs. 3(d)–3(f)]. These results indicate that the core size can be controlled by changing the core flow velocity, and that the microfiber-shaped tissue of over 50 *μ*m core diameter can be formed. Figure 3(h) shows the time transient of the diameter of the microfiber-shaped tissues during the culture, indicating that the diameter of the core slightly increased with cell proliferation.

**FIG. 4.**
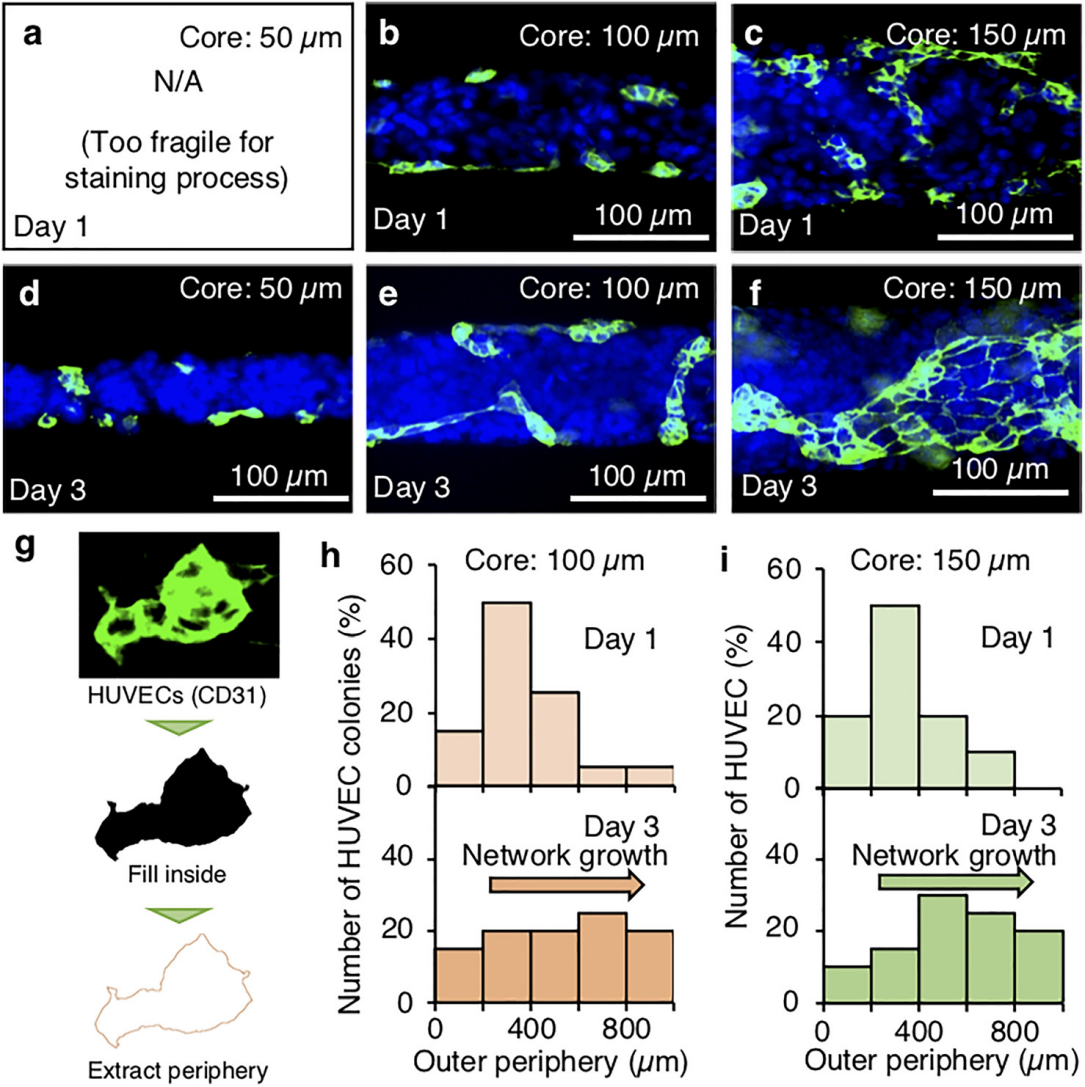
HUVEC networks with different core diameters. (a)–(f) Confocal fluorescent microscopic images of HUVECs (stained green) in the microfiber-shaped tissue (nuclei of Hep-G2 and HUVEC are stained blue). (g) Extraction of the length of the periphery. (h) and (i) Distribution histogram of the length of the periphery of the HUVEC networks of core diameter 100–150 *μ*m from day 1 to day 3 (*n *=* *20).

Figures 4(a)–4(f) show the confocal fluorescent images of immunostained HUVECs (CD31, green; nuclei, blue) in the microfiber-shaped tissues with different core diameters of the core (50 *μ*m, 100 *μ*m, and 150 *μ*m). Note that it was difficult to image the 50 *μ*m core tissue at day 1 because it was too fragile to be manipulated during the removal of the shell during the staining processes. The other microfiber-shaped tissues (core diameters: 100 *μ*m and 150 *μ*m) showed HUVEC colony formation on day 1 [Figs. 4(b) and 4(c)]. Although the HUVEC colonies were small in the microfiber with a 50-*μ*m core diameter on day 3 [Fig. 4(d)], a networklike expansion of the HUVEC colonies was observed, in both microfibers with 100- as well as 150-*μ*m core diameters [Figs. 4(e) and 4(f)]. Note that the microfiber-shaped tissue (core diameter: 50 *μ*m) after the removal of the shell collapsed [Fig. 4(a)], because the 50-*μ*m-diameter tissue was too thin and fragile to withstand the immunostaining procedure. To evaluate the expansion of HUVECs in the microfibers, we quantitatively measured the length of the periphery of the HUVEC networks and evaluated their growth from day 1 to day 3 [Figs. 4(g)–4(i)]. Analysis of the fluorescent images indicated that the networklike HUVEC colonies became longer upon culturing for 3 days.

### Ratio of HUVEC/Hep-G2 cells for HUVEC network formation

**FIG. 5.**
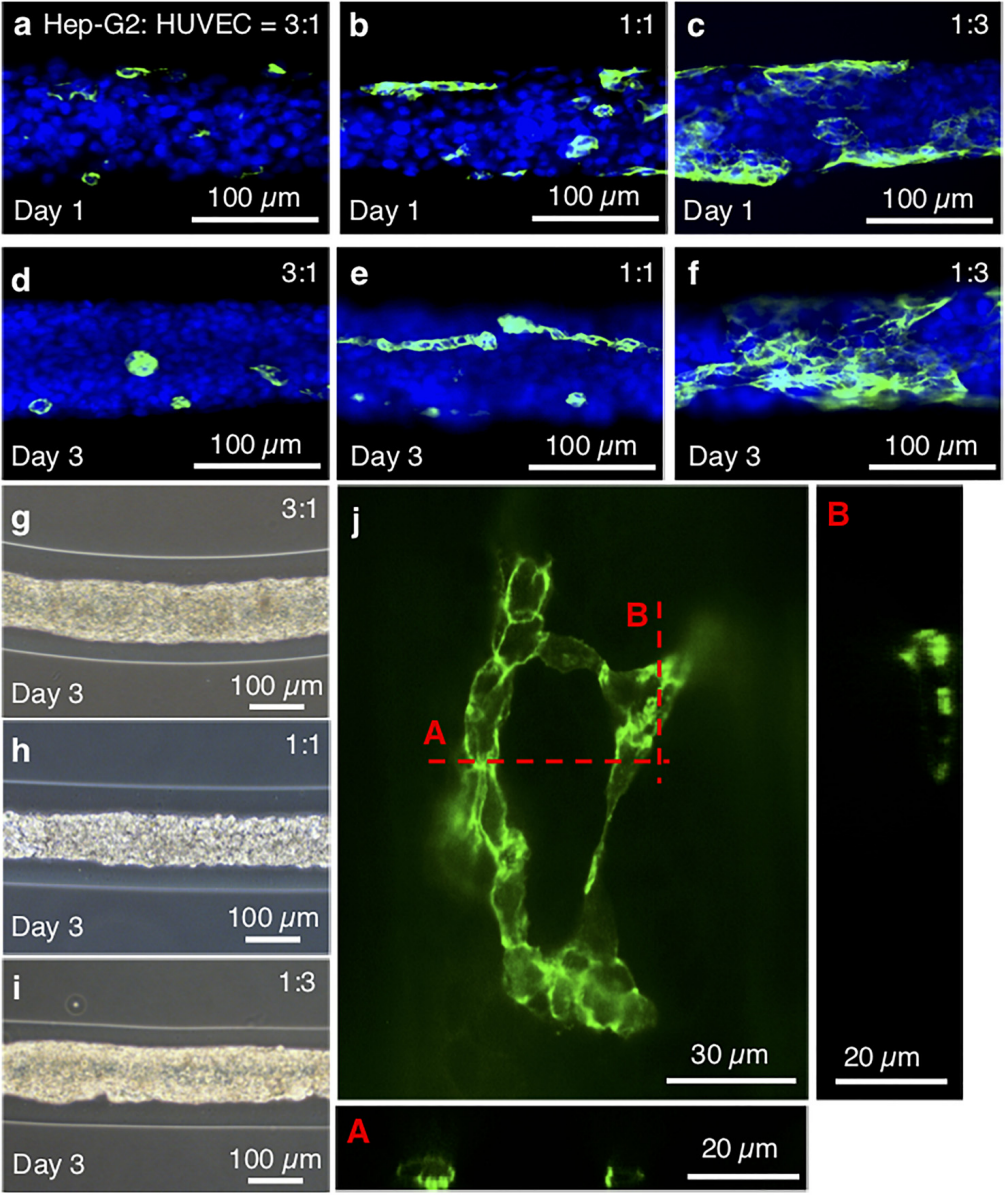
Observation of HUVEC networks with different Hep-G2/HUVEC ratios. (a)–(f) Fluorescence confocal microscopy images of HUVEC networks in the tissues where the cell ratios of Hep-G2:HUVEC were 3:1 (a) and (d), 1:1 (b) **and** (e), and 1:3 (c) and (f). (g)–(i) Phase contrast microscopy images of the microfiber-shaped tissues on 3 days. (j) Confocal image of the HUVEC networks in the microfiber-shaped tissue with a core diameter of 100 *μ*m and a 1:3 cell ratio.

In order to evaluate how the Hep-G2 cell/HUVEC blending ratio influences the formation of the networklike HUVECs, we fabricated microfiber-shaped tissues using three different Hep-G2:HUVEC blending ratios: 1:3, 1:1, and 3:1. Therefore, the ratio of HUVECs in all seeded cells was 25%, 50%, and 75% for equal intervals for figuring out the HUVEC network forming conditions. Phase contrast microscopy images of these three microfibers on day 1 and day 3 [Figs. 5(a)–5(f)] showed that, although there were no differences in their appearance on day 3 [Figs. 5(g)–5(i)], the sizes of the HUVEC networks in the different microfibers were different. HUVEC colonies did not expand in the 3:1 Hep-G2:HUVEC ratio microfiber, but networklike HUVEC connections were constructed in the 1:1 and 1:3 Hep-G2:HUVEC ratio microfibers. The HUVECs in the microfiber-shaped tissue were immunostained by CD31 as a functional marker. This marker shows the construction of endothelial networks^23^ to check the existence of the endothelial cell adhesion junctions. From the fluorescent image [Fig. 5(j) and supplementary Fig. 2], the cultured HUVECs expressed CD31 at the region of cell-cell contact, indicating the formation of cell-cell junctions for the platelet endothelial cell adhesion molecule-1 (PECAM-1). However, the fluorescence ratio of HUVECs is lower than 1:1 and 1:3. A reason is the change of the number of cells before and after the construction of the microfiber-shaped tissue. The other is that the CD31 selectively stains the junction of HUVEC networks. Actually, the ratio of Hep-G2 and GFP expressing HUVECs (1:1) in the microfiber-shaped tissue (supplementary Fig. 3) looks close to 1:1. The reconstructed cross-sectional images of HUVEC networks clearly show that tubelike tissues were formed by the HUVECs in the microfiber (core diameter: ∼100 *μ*m, Hep-G2:HUVEC = 1:3). This indicates that the endothelial networks were formed in the microfiber-shaped tissue.

### Construction of the macroscopic tissue assembly

Finally, construction of macroscopic tissues was demonstrated using microfiber-shaped tissues with HUVEC networks as building blocks. In order to show that various 3D assembled tissue shapes can be constructed, two basic macroscopic assemblies (a layerlike assembly and a wrappinglike assembly) for building a 3D assembled tissue were formed: a parallel tissue and a reeled tissue. A critical reason for assembling these macroscopic tissues using the microfiber-shaped tissues was that macroscopic tissues have cell-to-cell connections, whereas the shells of the microfiber-shaped tissues can be removed by enzymatic degradation.

**FIG. 6.**
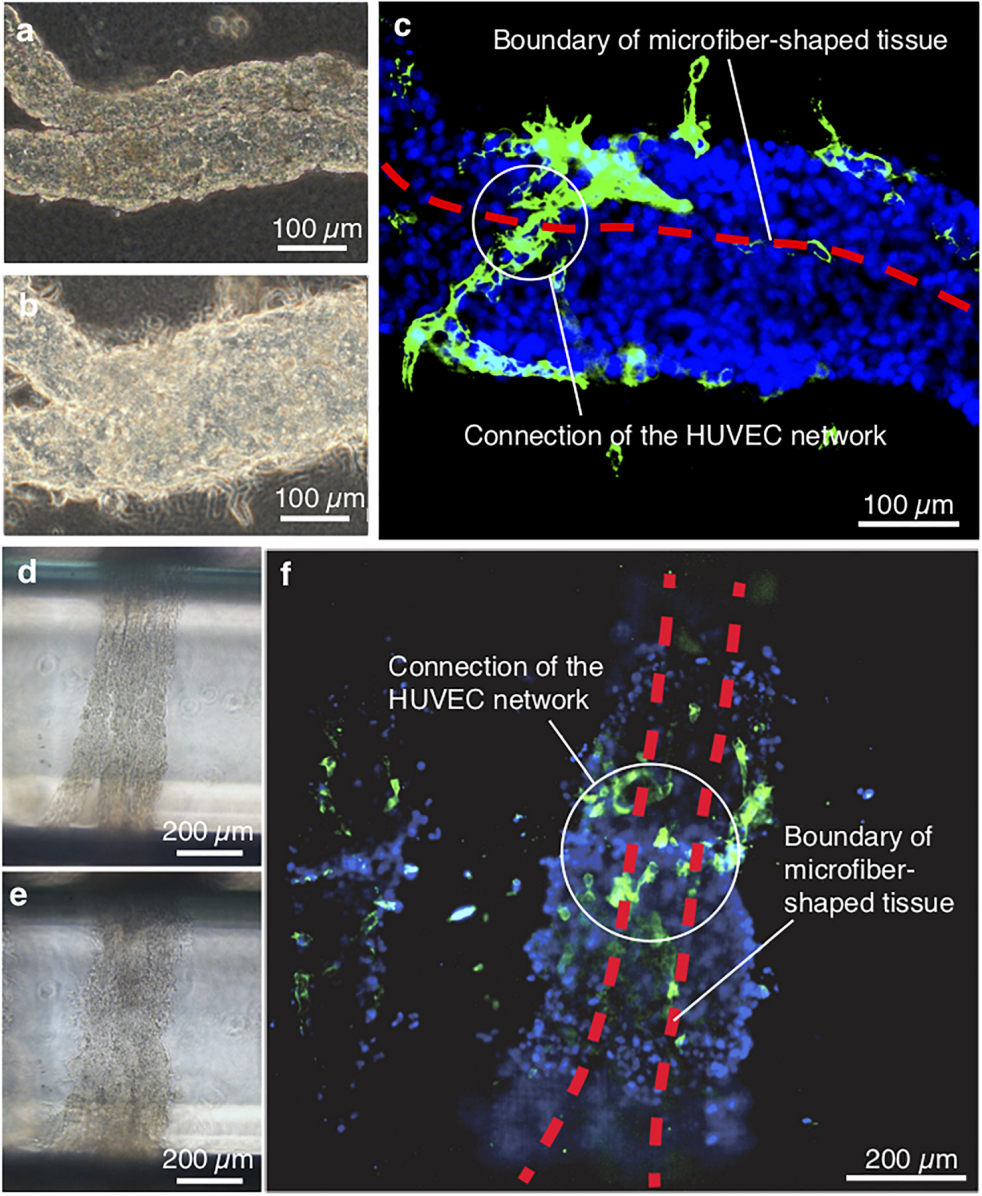
Macroscopic assemblies of the microfiber-shaped tissues. The microfiber-shaped parallel tissue (a)–(c) and the microfiber-shaped reeled tissue (d)–(f) were adjacently arranged in parallel and on the glass, respectively. The microfiber-shaped tissues (a) and (d) were connected to each other in parallel (b) or on the glass (e) after 2-day cultivation. Connections of the HUVEC networks between the microfiber-shaped tissues in both tissues (c) and (f) were observed by confocal microscopy.

The microfiber-shaped tissue was fold up and arranged in parallel. After that, the gaps between the microfiber-shaped tissue (diameter: 100 *μ*m, ratio of the cells 1:3) were shrunk and arranged in parallel on a dish and the alginate shell was removed by alginate lyase. By dissolving the shell after 15 min, the gaps between the assembled tissues were filled, as shown in Fig. 6(a). After an additional 2 days of culture, the parallelly arranged microfiber-shaped tissues connected to each other to form the macroscopic assembled tissue [Fig. 6(b)]. The confocal fluorescence microscopy image of the immunostained assembled tissue obviously indicated that the HUVEC networks between the microfiber-shaped tissues connected spontaneously [Fig. 6(c)]. Similarly, the microfiber-shaped tissues reeled on a glass tube [Fig. 6(d)] also connected to each other and a larger macroscopic tube-shaped tissue with HUVEC network connections was formed [Figs. 6(e) and 6(f)]. These results indicate that our microfiber-shaped tissue building block is capable of constructing a large macroscopic tissue with spread HUVEC networks.

## DISCUSSION

As for the cell composition of the building blocks, microfiber-shaped tissues in the core-shell hydrogel microfibers have been formed by using a single cell type.^16,22^ Microfibers containing multiple types of cells have been reported; for example, glial cells and neurons differentiated from neural stem cells^17,20^ and musclelike cells differentiated from dedifferentiated fat cells.^24^ Those differentiated cells were differentiated from a single type of stem cell encapsulated in the hydrogel microfibers. Therefore, our Hep-G2 microfiber-shaped tissues with HUVEC networks are a novel class of fiber-shaped building blocks containing heterogeneous cell types for the assembly of macroscopic tissues with endothelial network constructs.

In the tissue engineering field, the construction of artificial liver tissue that mimics the metabolism, detoxification, and secretion of bile *in vivo* has been eagerly researched for application in pharmacokinetic testing and regenerative medicines. The simplest method which exists currently involves the perfusion and culture of hepatocyte spheroids in a bioreactor using a hollow microfiber cartridge.^25^ It has been confirmed that hepatocyte functions, such as the albumin producing ability, can be maintained using such a method. However, this method is unable to maintain tissues that are less than 500 *μ*m in diameter for a long period.^24^ This leads to central necrosis in the hepatic spheroids because spheroids are analogous to avascular tissues or tumor masses and have a diffusion limitation of about 150–200 *μ*m for many molecules, particularly oxygen.^26^ Hence, hepatocyte spheroids are not suitable for the formation of tissues with a uniform cell quality. Similar to the spheroids, the thick microfiber-shaped tissue leads to central necrosis in malnutrition and lack of oxygen. On the other hand, the thin microfiber-shaped tissue after removal of the shell is fragile. However, through optimization of the core size of the collagen gel containing cells, the microfiber-shaped tissue in our study was able to structurally prevent the increase in the tissue size because the tissue was physically wrapped by an alginate shell. In the same manner, the microfiber-shaped tissue was able to retain the structure without collapse after removing the shell.

In the case of microfiber-shaped tissues, it is necessary to enclose cells in a shell of calcium alginate to generate the microfiber shape so that the cells do not spread outside and to ensure that the cells do not spill out. The shell of calcium alginate also contributes to enhancing the mechanical strength for the maintenance of the microfiber-shaped tissue.^16^ The calcium alginate hydrogel was mechanically strong, and the encapsulated cells did not leak even after one week [Fig. 2(c)]. However, the hydrogel shells covering the tissues prevent intertissue connections when the tissues are assembled on a macroscopic scale. An *in vitro* study on a microfiber-shaped liverlike tissue^27^ reported that hepatic tissue clusters were formed independently in the hydrogel microfibers. Once the shell of calcium alginate was removed, the strength of the microfiber-shaped tissue became fragile depending on the types of tissues.^16^ For this reason, it is difficult to assemble the microfiber-shaped tissues without the shell because the tissue without the shell was fragile. However, our approach can be used to construct connections between microfiber-shaped tissues as well as HUVEC networks (Fig. 6). In our method, a microfiber-shaped tissue with the alginate shell was assembled first, followed by dissolving the alginate shell with alginate lyase treatment. Then, the gap between the microfiber-shaped tissues was finally squeezed and filled manually. By using this method, the assembled tissue succeeded in constructing macroscopic tissues stably. The shape of the macroscopic tissue is not limited, and various shapes can be formed because of the good handleability of the microfiber. For example, parallel and reeled structures like woven structures,^16^ and bundled structures^28^ were presented in this work,. Thus, our microfiber-shaped tissue could be a promising building block for bottom-up tissue reconstruction with an endothelial network. We believe that our microfiber-based method could be applied to other tissues, such as the liver, kidney, and heart tissues. The microfiber-shaped building-block tissues could be used for *in vitro* tissue models in drug testing or implantable artificial organs for regenerative medicine, by combining with 96-well drug assay array systems or immunosuppression materials.

## METHODS

### Double coaxial flow microfluidic device

To fabricate the microfiber-shaped microscale tissue, we used a double coaxial microfluidic device based on a previously published work.^16^ This device was composed of glass capillaries and connectors made of resin. A glass capillary tube (outer diameter: 1.0 mm, inner diameter: 0.6 mm, G-1, Narishige, Tokyo, Japan) was sharpened using a tip-puller (P-10, Narishige); the tips of these glass capillaries were cut using a micro forge (EG-44, Narishige) and the tip diameter was adjusted to approximately 200 *μ*m. A square glass tube (outer diameter: 1.4 mm, inner diameter: 1.0 mm, 8100–100, VitroCom, NJ, USA) was used to fix the inner glass capillary tube. A connector was fabricated using a 3D printer (AGILISTA, Keyence, Osaka, Japan). Those glass capillaries and connectors were assembled on a slide glass (S2124, Matsunami Glass Ind., Ltd., Osaka, Japan). All inlets were connected to syringes via Teflon tubes (JR-T-082-M10, Shimadzu Corp., Kyoto, Japan). All syringes were connected to syringe pumps.

### Cell culture

For the formation of the microfiber-shaped endothelial hepatic tissue, we used two types of cells: (i) human hepatoma cells (Hep-G2) were purchased from the RIKEN Cell Bank (RCB1886, Ibaraki, Japan) and used for constructing the basic structure of the microfiber-shaped tissue. This cell line (passages 22–27) was maintained in Dulbecco's Modified Eagle Medium (DMEM, D5796–500 ML, Sigma-Aldrich, MO, USA) containing 10% (v/v) fetal bovine serum (FBS) and 1% (v/v) penicillin-streptomycin solution (P4458, Sigma-Aldrich). (ii) HUVECs (passage 3–5) were purchased from Lonza Walkersville, Inc. (C2519A, MD, USA) and used for constructing the internal endothelial network structure of the microfiber-shaped tissue. These cells were maintained in supplemented endothelial cell growth medium 2 (EGM-2, PromoCell, Heidelberg, Germany). All cells were maintained at 37 °C and 5% CO_2_ in humidified conditions.

### Formation of the microfiber-shaped tissue

A triple concentric laminar flow composed of core, shell, and sheath flows was created in the microfluidic device for the formation of the microfiber-shaped tissue. For the core flow, a cell suspension containing Hep-G2 and HUVEC (1.0 × 10^8^ cells/ml) cells in type-I collagen (4 mg/ml, derived from bovine dermis) (IAC-50, KOKEN Co. LTD., Tokyo, Japan) was prepared. For the shell flow, 1.5% (w/w) sodium alginate solution (194–13321, Wako, Osaka, Japan) dissolved in 145 mM sodium chloride (191–01665, Wako) was prepared and sterilized with a 0.22 *μ*m filter. For the sheath flow, 100 mM calcium chloride (090–00475, Wako) solution with 3% (w/w) sucrose (196–00015, Wako) was prepared and sterilized using an autoclave.

Microfiber formation was carried out at 4 °C to prevent the gelation of the collagen pregel solution. For sterilization, the microfluidic device was filled with 70% (v/v) ethanol for 20 min, followed by rinsing of the device with saline. After that, we performed the following steps: (1) the separated syringes were filled with the cell suspension for the core flow, with the sodium alginate solution for the shell flow, and with the CaCl_2_ solution for the sheath flow. (2) The syringe pumps sequentially started to inject the core flow (flow rate Q_core_ = 5–95 *μ*l/min), the shell flow (flow rate Q_shell_ = 55–95 *μ*l/min), and the sheath flow (flow rate Q_sheath_ = 2.5 ml/min). Since laminar flows were formed in the microfluidic device, each flow did not mix with the other. A microfiber containing the cells was formed in the microfluidic device and collected in a centrifuge tube filled with saline. (3) Once the microfiber of the desired length was formed, the flows were stopped. (4) The microfiber containing the cells in the centrifuge tube was transferred to a 100 mm culture dish. The saline in the dish was replaced with the mixed medium (DMEM:EGM-2 = 1:1). (5) The microfiber-shaped tissue in the dish was incubated at 37 °C to solidify the collagen at the core. Subsequently, the microfiber containing the cells was cultured at 37 °C and 5% CO_2_ in humidified conditions.

### Immunofluorescent staining

To visualize the construction of the HUVEC network, we stained microfiber-shaped hepatic tissues by immunofluorescent staining as follows. The microfiber-shaped tissue was fixed in 4% paraformaldehyde phosphate buffer solution (163–20145, Wako). During the fixation, the alginate shell was removed from the microfiber-shaped tissue. After 15 min of fixation, the tissue was permeabilized with 0.1% Triton-X100 (A16046, Alfa Aesar, MA, USA) in phosphate buffered saline (PBS, 163-25265, Wako) for 10 min and soaked in 1% bovine serum albumin (BSA, A2153, Sigma-Aldrich) in PBS to block nonspecific binding. Subsequently, the microfiber-shaped tissue was incubated with purified mouse antihuman CD31 (555444, BD Biosciences, NJ, USA) in PBS overnight. Next, the tissue was rinsed with PBS and incubated with Alexa Fluor 488-conjugated goat antimouse IgG secondary antibody (A-11001, Invitrogen, CA, USA) in PBS and DAPI (D1306, Invitrogen) for nucleus staining. After rinsing with PBS, the tissue was arranged on a 35-mm glass base dish (3961-035, IWAKI Co. LTD., Tokyo, Japan) and sealed with a mounting agent (Fluoromount/Plus, Diagnostic Biosystems, CA, USA).

### Macroscopic assembly of the microfiber-shaped tissue

We prepared endothelial microfiber-shaped tissues with a diameter of ∼100 *μ*m and a cell ratio of 1:3 (Hep-G2:HUVEC) for the two types of assemblies: parallel tissue and reeled tissue. For the parallel tissue, the microfiber-shaped tissue was cut to about 5 cm in a dish folded, and arranged using a pointed glass tube. After that, the medium was removed carefully, and the gaps between the microfiber-shaped tissues (diameter: 100 *μ*m, ratio of the cells 1:3) were shrunk and arranged in parallel on a dish. For the removal of the alginate shell, 40 *μ*g/ml alginate lyase (A1603, Sigma-Aldrich) was spread over the assembled tissue. By dissolving the shell after 15 min, the gaps between the assembled tissues were filled manually by using pulled glass tubes. After that, the arranged tissues were covered with a type-I collagen gel to fix the position. The arranged tissues were cultured in the medium for another 2 days.

For constructing the reeled tissue, a 5-cm-length microfiber-shaped tissue (diameter: ∼100 *μ*m, HepG2:HUVEC ratio = 1:3) was taken up in the air with a sterilized ø1 glass tube. The microfiber-shaped tissue was then reeled up by twirling the glass tube. After reeling, the glass tube with the microfiber-shaped tissue was put on the dish, and 40 *μ*g/ml alginate lyase (A1603, Sigma-Aldrich) was spread over the assembled tissue. After 15 min, the assembled tissue was lifted in the air and the gap between the assembled tissues was filled manually. The reeled-up microfiber-shaped tissue was covered with a type-I collagen gel. In the incubator, the reeled tissue was cultured for another 2 days.

### Observation and evaluation of the microfiber-shaped tissue and the assembled tissues

The microfiber-shaped tissue and the assembled tissues (the parallel tissue and the reeled tissue) were observed by using a phase contrast and fluorescence microscope (IX73, Olympus Corporation, Tokyo, Japan) and a confocal microscope (DSU, Olympus Corporation) equipped with a color CCD camera (Zyla 5.5, Oxford Instruments, Abingdon, UK) operated by cellSens (cellSens Standard 1.7, Olympus Corporation) and MetaMorph (MetaMorph Ver 7.8.8.0, Molecular Devices, CA, USA), respectively. Both the microfiber-shaped tissue and the assembled tissues were observed on a cell culture dish. To analyze the HUVEC network in the microfiber-shaped tissue, green color was extracted from the confocal microscopic images, and the length of the periphery was measured by ImageJ (National Institutes of Health, Bethesda, MD). In the length of the periphery, the HUVEC cluster stained with CD31 was extracted with image J. Upon staining with CD31, the endothelial cell adhesion junctions of HUVEC were observed by the fluorescence microscope. That is, the periphery of each HUVEC was extracted because the adhesion between cells was stained. Therefore, the inside of each HUVEC was filled and the length of the periphery of the HUVEC cluster was extracted. In order to evaluate the growth of the HUVEC network, Fig. 4 shows the distribution histogram by measuring the change in the size of the HUVEC network.

### Ethics approval

Ethical approval was not required for this study.

## SUPPLEMENTARY MATERIAL

See the supplementary material for the supplementary figures of the microfiber-shaped tissues.
